# Challenges in Formulation and Implementation of Hepatitis B Elimination Programs

**DOI:** 10.7759/cureus.14657

**Published:** 2021-04-24

**Authors:** Zaigham Abbas, Minaam Abbas

**Affiliations:** 1 Gastroenterology and Hepatology, Dr. Ziauddin University Hospital, Karachi, PAK; 2 Medicine, School of Clinical Medicine, University of Cambridge, Cambridge, GBR

**Keywords:** hepatitis b(hbv), who, elimination programs, vaccination, mother to child transmission, nosocomial

## Abstract

Nearly 257 million individuals have contracted hepatitis B infection around the world. However, only 10% of them know about their illness. Mother to child transmission, nosocomial spread, and sexual transmission are the major etiological factors. Finding the missing millions is a global issue. Hepatitis B care is more difficult compared to hepatitis C as not all patients require treatment and the selection of patients is not straightforward. To eliminate hepatitis B infection, the program should screen pregnant women and start antiviral therapy from the 28th week of pregnancy if hepatitis B virus (HBV) DNA≥ 200,000 IU/mL or hepatitis B e-antigen (HBeAg) reactive. Prevention of perinatal infection, birth dose and neonatal vaccination, post-vaccination monitoring of high-risk groups, catch-up vaccination, and registration of the carriers should be an integral part of the program. Continuum of care is important when planning the elimination program from addressing the risk factors, testing, and referral for treatment. The program should integrate test and treat hepatitis services with existing local health care services. There is a need to create the right environment, raise awareness, remove stigma, and increase screening of those at risk and manage those who require treatment. A national policy should be prepared for capacity building, fund allocation, and implementation strategies. Micro-elimination strategies should boost national elimination effects. Guidelines to diagnose and treat patients with hepatitis B should be simplified. Surveillance should be done to monitor progress, and determine the impact of the elimination program on incidence and mortality, and services.

## Introduction and background

Worldwide, about 257 million persons have been exposed to hepatitis B (2015 estimates). Only 10% of hepatitis B patients know about their disease [1]. If left untreated, about 25% of infected individuals will progress to cirrhosis or hepatocellular carcinoma (HCC) [1-3]. Every year, 900,000 patients die of hepatitis B and its complications. Around 58% of children globally do not have access to the birth dose of the hepatitis B vaccine. Unfortunately, as of 2017, only 28% of countries have interventions to support hepatitis B virus (HBV) elimination plans, citing cost as the primary impediment [1]. Coronavirus disease 2019 (COVID-19) has badly affected all the services related to hepatitis elimination [4]. Consequently, it seems difficult to achieve the WHO target of elimination of viral hepatitis as a major public health threat.

## Review

WHO goals for viral hepatitis elimination

In May 2016, the WHO Global Health assembly released its goals to eliminate viral hepatitis as a major public health threat by 2030 [5]. This included some important targets: 90% of infants should have a birth dose vaccination for hepatitis B, HBsAg (hepatitis B surface antigen) prevalence should be below 0.1% in children 5 years of age, 100% of blood donations should be screened for viral hepatitis (both B and C), and 90% of injections should be administered with safe syringes. For testing, 90% of individuals with viral hepatitis should be aware of their infection, and 80% of those infected should be treated. This would result in a 90% reduction in the incidence of new infections and a 65% reduction in mortality. However, according to the forecast HBsAg prevalence is not going to change assuming the status quo, many countries will only to able to achieve some of the set goals later than the year 2050. Only three countries are expected to achieve a 90% reduction in HBV incidence, none will achieve a 65% reduction in mortality relative to 2015, and no country will achieve all the current HBV elimination targets [6].

Formulating an elimination program

When formulating a hepatitis B elimination program, we should know about the disease burden to deal with. What are the risk factors? What are the targets? Numbers needed to screen and treat? The capacity and cost issues, policy, guidelines, financing, and implementation strategies should be kept in mind. The program should focus on prevention, which means safe blood and infection control, vaccinations, harm reduction measures for individuals participating in high-risk activities, and prevention of mother-to-child transmission. The facilities for testing and initiation of therapy among those already infected should be expanded. Diagnosis and treatment are really important when striving toward elimination. Barriers to the effective implementation of hepatitis control programs should be preempted and properly addressed (Table 1) [7-9].

**Table 1:** Barriers to effective implementation of hepatitis control programs in the developing countries

Lack of diagnostic and treatment capacity
Scarce and expensive medicines
Lack of dissemination of information via media involvement
Poor routine immunization
No birth dose vaccine
Lack of routine maternal screening
Unawareness about treating hepatitis B during pregnancy
Poor compliance to follow the schedule of 0,1 & 6 months for hepatitis B vaccine
A disconnect between duty bearers and right holders
Lack of coordination among public /private and private/private players
Low political priority
Lack of funding from government and donor agencies
The large increase in hepatitis due to the persistence of risk factors and new case findings
An outdated health care system
Lack of awareness/education both on the supply side (health care providers) and demand-side (community)
Lack of trained specialists and health workers
Quackery
COVID-19 pandemic

Risk factors for HBV infections

Worldwide mother-to-child transmission is a prime risk factor [10]. Sexual transmission also exists. The nosocomial spread is still a major issue in developing and poor countries. Multiple therapeutic injections, unsafe blood transfusions, reuse or sharing of needles and syringes, surgical and dental procedures, and needle-stick injuries in healthcare workers, all are contributing factors [11]. All these healthcare-related issues are modifiable and policies and procedures are needed to be developed and enforced to ensure that the transmission of HBV is halted.

Find the missing millions

Finding the missing millions is a global issue. The infection is hugely under-diagnosed and under-treated even in the most developed countries [12]. About 80% of carriers of hepatitis B and C are undiagnosed. What are the reasons for missing millions of infected people with hepatitis B? We are not doing the mass screening. It is of low political priority. There is a lack of sufficient knowledge about hepatitis B and C and risk factors. Those at risk do not demand to screen as they are asymptomatic and feeling well or ascribe their symptoms to other things, many of these hail from underprivileged communities, and stigma and discrimination after diagnosis of hepatitis B exist.

Family doctors take little action to motivate their patients to get tested for hepatitis B and C [13]. They may be too busy or less motivated for case-finding. There may be a lack of knowledge of symptoms until end-stage liver disease with complications occurs. Due to a lack of understanding of abnormal liver function tests (LFTs), they dismiss subtle changes. A disparity exists between urban and rural populations in terms of physician’s approach, access to diagnostics, and treatment.

Case finding and therapy is cost-effective

It has been shown that case-finding and therapy for chronic viral hepatitis in primary care are potentially difficult with community-based interventions even in developed countries [13]. However, screening for viral hepatitis in primary care by incentivized practitioners appears cost-effective and helps to identify a cohort of motivated individuals who will engage with treatment. A study done on HBsAg prevalence in the Gambia based on the Markov state transition model confirms that community-based screening and treatment for chronic hepatitis B (CHB) is cost-effective in regions with an intermediate and high prevalence of hepatitis B [14]. A population-based, cluster-randomized, controlled trial between 1985 and 1990 from China confirms that the cumulative incidence probability of HCC and cumulative mortality probability of liver diseases in the vaccination group is much less compared to the control group [15]. Seroprevalence of hepatitis B virus in Taiwan 30 years after the commencement of the national vaccination program has decreased to less than 1% in university students which used to be about 10% [16]. The strategy to completely eradicate hepatitis B infection in Taiwan included general preventive hygiene caution measures and an effective anti-viral therapy if indicated, screening of pregnant women, perinatal infection prevention, neonatal vaccination, post-vaccination monitoring of high-risk groups, and registration of carriers and management adequately [17].

A systematic review addressed hepatitis B screening with economic evaluations in low- and middle-income countries (LMIC). Nine studies fulfilled the eligibility criteria. Screening with ‘catch-up’ vaccination for younger adults yielded benefits above costs, and screening linked with treatment showed cost-effectiveness that might be affordable for some LMICs [18].

WHO 2020 recommendations: pregnant women

Recently, on World Hepatitis Day, WHO released its guidelines to prevent mother-to-child transmission of hepatitis B [19]. Pregnant women testing positive for HBV infection with an HBV DNA≥ 200,000 IU/mL should receive tenofovir prophylaxis from the 28th week of pregnancy until at least birth, to prevent mother-to-child transmission of HBV. This is in addition to three-dose hepatitis B vaccination in all infants, including a timely birth dose. If HBV DNA testing is not available, hepatitis B e-antigen (HBeAg) testing can be used as an alternative to HBV DNA testing to determine eligibility for tenofovir prophylaxis. The systematic reviews of antiviral therapy during pregnancy to prevent mother-to-child transmission (MTCT) favour treatment [19,20]

WHO suggests an Incremental approach to the prevention of HBV infection at birth and in the first years of life it includes maternal antiviral prophylaxis as mentioned above, HBsAg testing linkage to care and follow-up of infants, when available hepatitis B immunoglobulin for infants born to HBsAg and HBeAg positive mothers, and at least three doses of hepatitis B vaccine, including a birth dose within 24 hours.

Treatment of hepatitis B in elimination programs

HBV care is more difficult compared to HCV (Table 2).

**Table 2:** Differences between hepatitis C and B elimination PCR, polymerase chain reaction

	Hepatitis C	Hepatitis B
Vaccination	No vaccine	Birth dose vaccine a real challenge
Mother to child transmission	Not a major issue	A real threat
Diagnostic tests	Accessible and cheaper	Cost barriers
Treatment	Curable	Treatable
Eligible for treatment	All PCR positive patients	Selected patients
Drugs to treat	Cheap generics available	Issue in some countries
New drugs development	Occurred at a rapid pace	Slow
Awareness, advocacy	Increased after availability of directly acting antivirals (DAAs)	Community movements lacking
Monitoring and follow-up	Easy	Difficult with many steps

**Figure 1:**
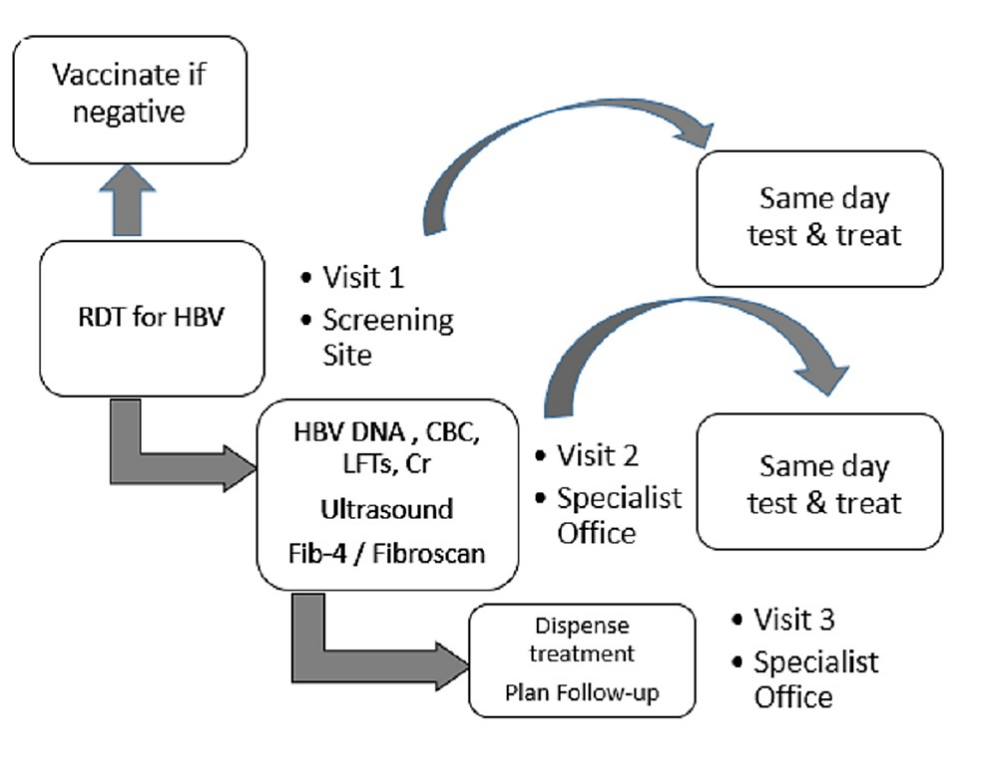
How to decentralize and simplify the service delivery in the hepatitis B elimination program RDT, rapid diagnostic tests; HBV, hepatitis B virus; DNA, deoxyribonucleic acid; CBC, complete blood counts; LFTs, liver function tests; Cr, creatinine; Fib-4, fibrosis-4

Continuum of care is important when planning the elimination program from testing to referral and treatment [7]. Hepatitis control programs should focus on training and educating staff to follow algorithms to deliver initial care. As not all patients require treatment for hepatitis B, the selection of patients is not straightforward in the case of hepatitis B. Patients who fulfil the eligibility criteria should be selected for treatment. Proper training of primary care physicians may minimize the requirements for specialist care and the number of visits as proposed in Figure 1.

The program should integrate services for managing viral hepatitis with existing health care services and work on improving safety, e.g., screening of blood products, provision of clean needles, and infection control in healthcare facilities. With the availability of generic drugs to treat hepatitis B, the cost of treatment has decreased drastically. Treatment with nucleoside analogues is safe and effective.

Micro-elimination strategies of hepatitis B

Micro-elimination strategies target individual population segments for which treatment and prevention interventions can be delivered quicker and more efficiently. These programs may address districts with high prevalence, identify pockets of high prevalence within districts, take measures to prevent transmission or address risk factors, and identify target groups to treat infection and prevent transmission. For example, these programs may focus on antenatal screening, infant vaccination, catch-up vaccination, vaccination of persons who inject drugs, prisoners, decompensated cirrhosis, veterans, or patients with haemophilia and homosexuals. It has been shown by creating a lifetime Markov model that strategies to vaccinate, prevent or treat CHB in high-risk populations result in a significant reduction in cirrhosis, decompensation, liver cancer, and chronic hepatitis death with the intervention compared to no intervention [21].

Micro-elimination strategies are tailored with realistic and well-defined targets and goals. These are pragmatic, time to achievement is shorter and cost can be predicted. Micro-elimination projects may generate a template in a small geographically defined population which may then be used to model services for larger intervention programs. Successful micro-elimination efforts encourage further public health strategies. Micro-elimination of hepatitis B, appear cost-effective and will have a positive impact on long-term outcomes with screen and treat or vaccinate strategy compared with no intervention.

Lessons from the successful Egyptian program

Egypt is running a very successful hepatitis control program and is achieving the WHO set targets [22,23]. All hospitals and rural health centres were involved. A screening centre was set up in every village. Mobile units were made available. A huge media campaign was started through TV, radio, social media, paper media, and SMS (short message service). A successful awareness campaign was run and society pressure generated. A person could walk into any screening centre. Rapid diagnostic tests were made available instead of ELISA (enzyme-linked immunosorbent assay). Seropositive patients were immediately referred to an evaluation centre. Free investigations and treatment were available. Call centres were set up to prevent dropouts and contact no-shows. The management guidelines were made simple. The focus was to decrease the number of visits. There was a continuous political will and support. The government allocated sufficient resources and funds to initiate and maintain the program. An empowered central decision-making body was set up.

How to scale up elimination and meet the targets

Public Health is a great issue in low-income countries where the cost of diagnostic tests is high keeping in mind that due to the unavailability of government facilities 70% of the population visits the private sector for health issues. Around 60-70% of expenditure is out of pocket. It is impossible to control hepatitis without the availability of free tests and treatment facilities. Guidelines to diagnose and treat patients with hepatitis B are to be made simplified, infrastructure to test and treat should be expanded and targets should be set up to test and treat in local health care centres. Sufficient funds should be allocated ad Innovative financing strategies should be devised to raise the money [7,11,24,25].

Validation of elimination efforts

Surveillance should be done to monitor progress, and determine the impact of the elimination program on incidence and mortality, and services. Monitoring may be done by conducting two surveys of national or high-risk groups a minimum of one year apart and estimate incidence between the two by age group [26]. There is a need to establish a national registry for decompensated cirrhosis and HCC linked to patient and death registries. Modelling may be used to estimate incidence when doing surveys is not possible.

## Conclusions

There is a high prevalence of HBV in many regions of the world. The disease burden will not decrease through 2030 with the continuation of the base strategy. Control of hepatitis B is feasible in the next 10 years or so through an aggressive national strategy that requires political will, strategic plans with timelines, multiple stakeholders’ involvement, strong infrastructure, public awareness, and funding (domestic and international resources). For harm reduction, a broad education campaign, control of nosocomial infections, treatment of pregnant ladies with high viral load, and vaccination including birth dose vaccination is warranted. A scale-up in screening is required to keep pace with the increase in treatment. A significant increase in the number of individuals being treated will mitigate the disease burden. Access to therapy is important. The availability of generics would make this endeavour more effective.
